# Systemic inflammation–based hematological indices and 90-day functional outcomes after intravenous thrombolysis in acute ischemic stroke: a systematic review

**DOI:** 10.3389/fneur.2025.1699939

**Published:** 2025-11-26

**Authors:** Huiying Huang, Weijun Wang, Qing Ma, Kun Cao

**Affiliations:** 1 Department of Neurology, People's Hospital of Leshan, Leshan, Sichuan, China; 2 Department of Neurology, People's Hospital of Daying County, Suining, Sichuan, China

**Keywords:** acute ischemic stroke, intravenous thrombolysis, systemic inflammatory response, composite inflammation indices, prognostic evaluation

## Abstract

**Background:**

Acute ischemic stroke (AIS) is one of the leading causes of mortality and long-term disability worldwide. Intravenous thrombolysis (IVT) with recombinant tissue plasminogen activator (rt-PA) remains the standard treatment for eligible patients; however, considerable inter-individual variability exists in post-treatment functional outcomes. Increasing evidence suggests that systemic inflammation plays a crucial regulatory role in both ischemic injury cascades and reperfusion efficacy. In recent years, several inflammation-based hematological indices derived from complete blood counts—such as the neutrophil-to-lymphocyte ratio (NLR), systemic immune-inflammation index (SII), systemic inflammation response index (SIRI), inflammation prognostic index (IPI), and pan-immune-inflammation value (PIV)—have been proposed. These indices comprehensively reflect the balance between innate immune activation and adaptive immune suppression and are considered potential prognostic biomarkers.

**Methods:**

Following the Preferred Reporting Items for Systematic Reviews and Meta-Analyses (PRISMA) guidelines, we systematically searched PubMed, Embase, and Web of Science for English-language studies published between 2015 and 2025 investigating the relationship between inflammation-based hematological indices and functional outcomes in adult AIS patients receiving intravenous rt-PA. Eligible studies were limited to IVT-only cohorts reporting associations between composite inflammatory indices and clinical outcomes. The search strategy was framed using the PICO (Population, Intervention, Comparison, and Outcome) approach, and study quality was assessed using the Newcastle–Ottawa Scale (NOS).

**Results:**

A total of 15 observational cohort studies involving approximately 4,000 AIS patients were included. Higher baseline or early values of NLR, SII, SIRI, and PIV were independently associated with unfavorable 90-day functional outcomes, with predictive performance (AUC) generally ranging from 0.70 to 0.80. Several studies further indicated that dynamic changes in inflammatory indices within 24–48 h after IVT provided stronger prognostic discrimination than baseline measurements, underscoring the clinical value of early immune monitoring during the acute phase of stroke.

**Conclusion:**

Systemic inflammation plays a central role in the pathophysiology and therapeutic response of AIS. Composite inflammation-based hematological indices are simple, economical, and reproducible tools that may assist in early risk stratification and individualized prognostic assessment following IVT. Future studies should incorporate dynamic longitudinal monitoring and integrate multimodal clinical and biomarker data within large, multicenter cohorts to improve model precision and enhance translational applicability.

## 1 Introduction

Cerebral ischemic stroke, also known as ischemic cerebral infarction, is a clinical syndrome caused by localized cerebral hypoperfusion leading to ischemia, hypoxia, and necrosis or softening of brain tissue, which manifests as acute focal or diffuse neurological deficits (1). In China, acute ischemic stroke (AIS) accounts for approximately 69.6–72.8% of all new stroke cases (2, 3) and is associated with high incidence, mortality, disability, and recurrence rates. According to the Global Burden of Disease Study, the incidence of AIS continues to rise in developing countries, particularly in China, where both incidence and disability rates remain among the highest worldwide (2). Although the overall incidence in Europe and North America has declined, the proportion of young adults affected by AIS has shown an upward trend (4).

Intravenous thrombolysis (IVT) remains one of the most effective acute-phase treatments for AIS, with a standard therapeutic window of 4.5 h; under perfusion-based imaging evaluation, the window may be extended to 9 h in selected patients (5). Intravenous alteplase is currently the standard thrombolytic agent in clinical practice, while tenecteplase has recently been explored as a potential alternative, particularly in specific patient subgroups (6, 7). Despite the proven efficacy of IVT in acute ischemic stroke, substantial inter-individual variability in clinical outcomes persists—partly driven by patients’ immune–inflammatory status (8). Therefore, identifying objective biomarkers that reflect the intensity of systemic inflammatory and immune responses is of great importance for risk assessment and individualized management.

Inflammatory responses play a pivotal role in the pathophysiology of AIS (9, 10). Multiple immune cell types contribute to ischemic brain injury: post-stroke immunosuppression increases the risk of secondary infections, while rt-PA-based thrombolysis may alter leukocyte activation and migration, promote neutrophil degranulation, and enhance blood–brain barrier (BBB) permeability, thereby influencing reperfusion outcomes (11, 12). Recently, several composite inflammation indices derived from hematological parameters have been introduced, such as the NLR-based SII, SIRI, IPI, and PIV. By integrating multiple immune cell parameters, these indices provide a more comprehensive representation of systemic inflammatory and immune balance and have emerged as promising prognostic predictors in AIS.

However, current studies predominantly focus on static baseline measurements and pay insufficient attention to dynamic in-hospital changes in these indices. Moreover, most clinical studies have investigated only a single marker (such as NLR), with limited systematic evaluation of composite indices. Considerable heterogeneity in inclusion criteria, treatment regimens, and outcome definitions across studies further contributes to inconsistent findings.

Accordingly, this review aims to systematically summarize current evidence regarding the association of multiple composite inflammatory indices (SII, SIRI, IPI, and PIV) with short-term outcomes in AIS patients treated with intravenous alteplase. We discuss their prognostic significance, methodological limitations, and future research directions.

## 2 Methods

### 2.1 Search strategy

Literature searches were conducted in three databases: PubMed, Web of Science, and Embase. Reporting followed the PRISMA (Preferred Reporting Items for Systematic Reviews and Meta-Analyses) statement and checklist (13). We searched for all articles identified and published from 2015 through October 2025 using the following keywords and controlled terms (including synonyms and exploded terms where applicable): A reproducible search strategy was developed with MeSH/Emtree terms and free-text keywords. For PubMed, the full string was: ((“Stroke, Ischemic”[Mesh] OR ischemic stroke*[tiab] OR ischaemic stroke*[tiab] …) AND (intravenous thromboly*[tiab] OR thromboly*[tiab] OR alteplase[tiab] OR rt-PA[tiab] OR recombinant tissue plasminogen activator[tiab] OR tenecteplase[tiab])) AND (inflammat*[tiab] OR “Inflammation”[Mesh])).

### 2.2 Study framework

This study aimed to determine whether complete-blood-count–based systemic inflammatory indices are associated with functional outcomes in adults with acute ischemic stroke (AIS) receiving intravenous thrombolysis. The research design adhered to the PICO (Population, Intervention, Comparison, and Outcome) framework (14, 15).

#### 2.2.1 Population

Adult AIS patients who received intravenous alteplase within 4.5 h of symptom onset. We excluded patients with active infection, autoimmune disease, malignancy, or other overt inflammatory conditions.

#### 2.2.2 Intervention/exposure

Measurement of systemic inflammation–related hematological indices before and/or after thrombolysis, including NLR, platelet-to-lymphocyte ratio (PLR), SII, SIRI, IPI, PIV.

#### 2.2.3 Comparison

Differences in clinical outcomes between high- versus low-inflammation groups; prognostic value across different time points (dynamic trajectories); and head-to-head comparisons among indices.

#### 2.2.4 Outcomes

The primary outcome was functional recovery at 3 months. Secondary outcomes included early neurological improvement, mortality, and symptomatic intracranial hemorrhage.

### 2.3 Eligibility and inclusion criteria

We included only original studies published in peer-reviewed English-language journals and excluded gray literature (books, conference abstracts, theses, preprints, notes, retractions) and review articles. All disciplines were considered at the initial screening stage; however, studies were required to explicitly report on adult AIS patients treated with intravenous rt-PA and to analyze the relationship between inflammation-based hematological indices and prognosis. During screening, duplicate records retrieved from the three databases were removed first, followed by title/abstract screening, and then full-text assessment and data extraction for eligible studies.

### 2.4 Study selection process

Screening was conducted in two stages. Stage 1 evaluated topic relevance based on titles, abstracts, and keywords to ensure alignment with the research question and search strategy. Stage 2 involved full-text review of preliminarily eligible articles to confirm adherence to inclusion and exclusion criteria. All included studies underwent independent dual review; disagreements were resolved by discussion with a third reviewer.

### 2.5 Quality assessment

The methodological quality and risk of bias of the 15 included observational cohort studies were evaluated using the Newcastle–Ottawa Scale (NOS) (16). This tool assesses three domains—selection (4 items), comparability (2 items), and outcome (3 items)—with a maximum score of nine stars. Most studies were rated as moderate quality (5–6/9), while seven achieved high quality (7–8/9). The main potential sources of bias arose from the non-randomized design and incomplete adjustment for confounding factors, whereas outcome assessment and follow-up were generally adequate and reliable (Table 1).

**Table 1:** Risk of bias assessment of the 21 included observational studies (Newcastle–Ottawa scale).

Study	Design	Selection (0–4)	Comparability (0–2)	Outcome (0–3)	Total (/9)	Quality level	Key justification
Chu et al. (36)	Retrospective single-center observational	3	1	3	7	High	Consecutive mild AIS patients (NIHSS ≤ 5); objective biomarkers (SIRI, SII); 3-month mRS outcome; multivariate adjustment for NIHSS, ISVS/ISVO, age, diabetes; complete follow-up.
Wu and Chen (32)	Retrospective cohort (INTRECIS registry)	3	2	2	7	High	Multi-center registry data (INTRECIS); dynamic NLR measurements (admission/24 h/12 d); 3-month mRS and mortality outcomes; multivariate adjustment for age, NIHSS, BMI, AF, HR, TOAST; complete follow-up.
Li et al. (33)	Retrospective single-center observational	3	1	2	6	Moderate	Clear inclusion/exclusion; objective biomarkers (NLR, LMR) at four time points; in-hospital outcomes (ENI, discharge mRS); limited confounder adjustment; small sample size (n = 102); short-term follow-up only.
Xu et al. (61)	Retrospective single-center observational	3	1	2	6	Moderate	Consecutive AIS patients with IVT (n = 286); PLR measured within 24 h; 3-month mRS follow-up; logistic regression adjusted for age, NIHSS, and stroke history only; no external validation; follow-up by phone without blinding.
Ma et al. (45)	Retrospective single-center observational	3	2	2	7	High	Clear inclusion/exclusion; objective biomarkers (SIRI, SII, IPI); 3-month mRS outcome; multivariate adjustment for age, NIHSS, glucose, TOAST, comorbidities; complete follow-up.
Wang et al. (47)	Retrospective single-center observational	3	1	2	6	Moderate	Retrospective single-center; objective biomarkers (PIV, SII, NLR, PLR); 3-month mRS; partial multivariate adjustment; no external validation; limited discussion of loss to follow-up and residual confounding.
Zhou et al. (62)	Retrospective single-center	3	2	2	7	high	Clear inclusion/exclusion; objective biomarkers (SII, NLR, PLR); 3-month mRS; multivariate adjustment for NIHSS, glucose, age, AF, diabetes, ASPECTS.
Zhao et al. (63)	Retrospective single-center observational	3	2	2	7	High	Consecutive AIS patients receiving IVT (n = 281); clear inclusion/exclusion; objective biomarkers (PT, SIRI, SII); 3-month mRS; multivariate adjustment for vascular risk factors, NIHSS, BP, glucose, TOAST; complete follow-up.
Pektezel et al. (64)	Retrospective single-center observational	2	1	2	5	Moderate	Retrospective design; single-center (n = 142); clearly defined IVT-only cohort; objective biomarkers (NLR pre- and post-IVT); limited multivariate adjustment (only age, HTN, NIHSS); no control for infection or comorbidities; 3-month mRS outcome reported but not blinded or externally validated.
Cheng et al. (65)	Prospective single-center observational	3	1	2	6	Moderate	Consecutive AIS patients treated with IVT (n = 381); clear inclusion/exclusion; 3-month mRS outcome; limited confounder adjustment (no dynamic biomarkers, limited comorbidity control); single-center design; no external validation or blinding.
Deng et al. (66)	Retrospective single-center observational	3	1	2	6	Moderate	Single-center retrospective; clear inclusion/exclusion; objective biomarkers (NPAR, NLR, PLR); 90-day mRS; multivariate logistic regression with limited confounder adjustment; no blinding or external validation.
Li et al. (38)	Retrospective single-center observational	3	1	2	6	Moderate	Retrospective design (2019–2022, n = 762); clear inclusion/exclusion; objective inflammatory markers (PLR, NLR, LMR, SII, SIRI, PIV); 3-month mRS outcome; partial multivariate adjustment (sex, age, glucose, NIHSS, comorbidities); no blinding, no external validation.
Weng et al. (41)	Retrospective single-center observational	3	1	3	7	High	Retrospective single-center cohort (n = 216); objective biomarker (SII); 3-month mRS follow-up; logistic regression with limited adjustment (age, smoking, AF, prior stroke, NIHSS); no blinding; no validation cohort; small sample.
Ma et al. (44)	Prospective single-center observational	3	1	1	5	Moderate	Prospective design with clear criteria and objective biochemical indices (SIRI, IPI) but small sample (n = 63), single-center, limited confounder adjustment and no blinding or external validation.
Chen et al. (18)	Retrospective single-center cohort	3	1	2	6	Moderate	Retrospective single-center (n = 161); objective lab index (SIRI); 3-month mRS outcome; logistic regression with limited covariate control; no blinding or validation; possible selection and confounding bias.

## 3 Results

**Figure 1:**
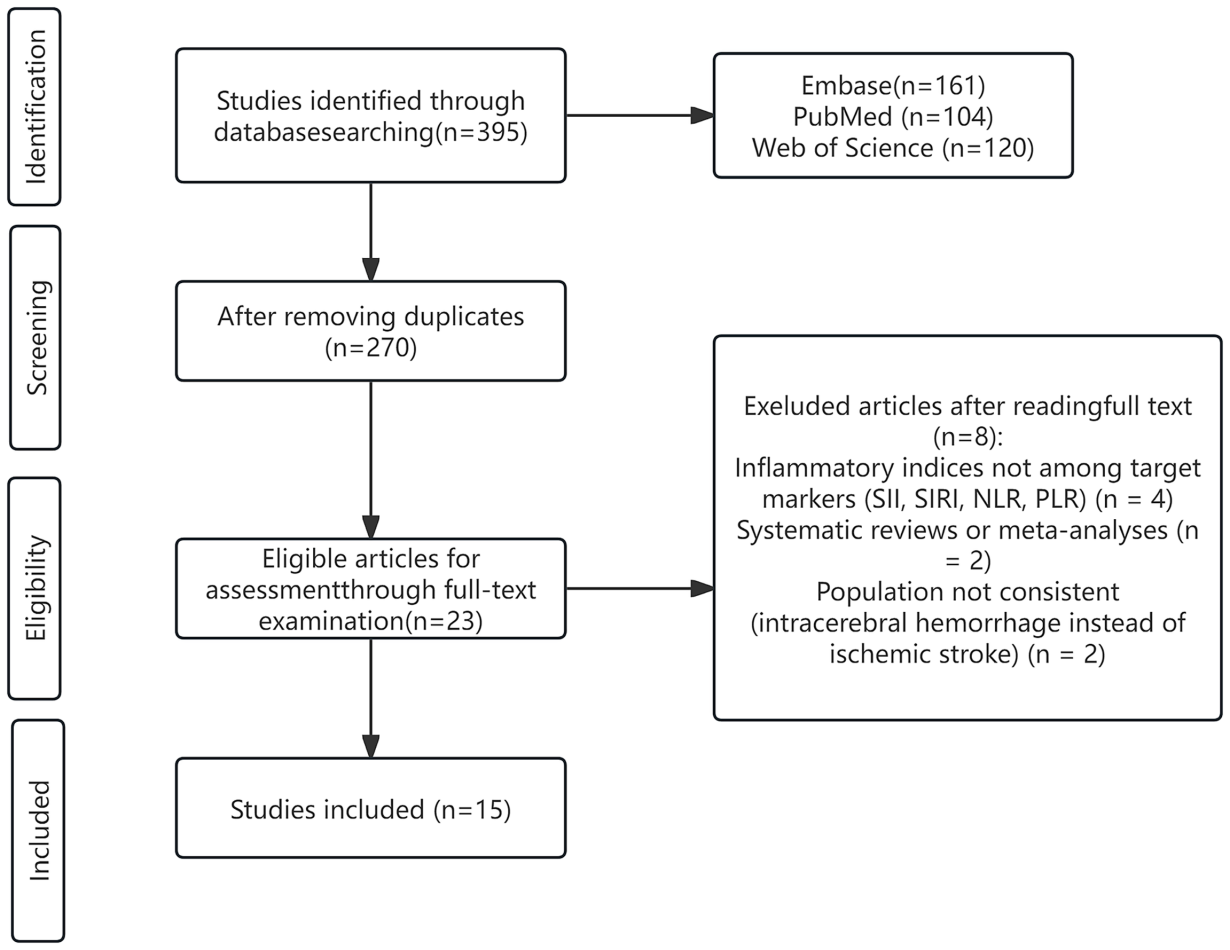
PRISMA flow diagram.

From the PubMed, Embase, and Web of Science databases, a total of 395 records were initially identified. After removing duplicates, 270 articles remained for title and abstract screening. Following the first-round evaluation, 23 articles underwent full-text review, among which 8 were excluded for not meeting inclusion criteria. Ultimately, 15 observational cohort studies were included for quantitative and qualitative synthesis (Figure 1).

These 15 studies (summarized in Table 2) collectively involved approximately 4,000 patients with acute ischemic stroke (AIS) who received intravenous thrombolysis with recombinant tissue-type plasminogen activator (rt-PA). Most studies adopted a single-center, retrospective design, with study periods spanning 2019 to 2025 and populations predominantly recruited from Chinese stroke centers. The inflammation-related hematological indices evaluated included: NLR, PLR, lymphocyte-to-monocyte ratio (LMR), SII, SIRI, IPI, PIV, and neutrophil-to-albumin ratio (NPAR). Among these, NLR, SII, and SIRI were the most frequently investigated indices. Overall, all included studies consistently demonstrated that higher baseline or early inflammatory index levels were significantly associated with unfavorable 90-day functional outcomes, defined as modified Rankin Scale (mRS) ≥ 2 or 3. The predictive performance of these indices ranged from moderate to good, with AUC values typically between 0.70 and 0.80. Several studies further reported that composite indices (e.g., SII and SIRI) achieved superior predictive accuracy compared with single-parameter ratios such as NLR and PLR, suggesting that integrating multiple immune-cell parameters may better capture the intensity of systemic inflammation. In addition, multiple investigations found that dynamic changes in inflammatory indices within 24–48 h after thrombolysis provided stronger prognostic discrimination than baseline measurements, lending further support to the concept of dynamic immune monitoring during the acute phase of AIS.

**Table 2:** Characteristics of included studies (according to reviewer recommendations).

Author (Year)	Country/Region	Sample size (n)	Study design	Therapy type	Inflammatory index	Index formula/definition	Cut-off (unit)	Timepoint (s) collected	Primary outcome (follow-up time)	Effect size (AUC/OR/HR [95% CI])	Key findings/Main results	Quality assessment/Risk of bias	Source
Chu et al. (36)	China (Shanghai)	240	Retrospective	IVT (rt-PA)	SIRI, SII	SIRI = NEU × MON/LYM; SII = PLT × NEU/LYM	SIRI = 1.00 × 10^9^/L	Baseline (pre-IVT)	mRS ≥ 2 @ 3 months	SIRI OR 2.938 (< 0.001); AUC 0.714; SII not significant (*p* = 0.918); Combined model AUC 0.773 (0.0017 vs. clinical factors alone)	Higher SIRI independently predicted unfavorable 3-month outcome in mild AIS; SII not significant after adjustment	high	*Front Neurol*, 2023
Wu and Chen (32)	China (Shenyang)	259	Retrospective	IVT(rt-PA)	NLR (dynamic)	NEU/LYM;cNLR₁–₂ = (NLR₂ − NLR₁)/NLR₁ × 100%; cNLR₁–₃ = (NLR₃ − NLR₁)/NLR₁ × 100%	NLR₂ ≥ 4.91; NLR₃ ≥ 3.94; cNLR₁–₂ ≥ 0.68; cNLR₁–₃ ≥ 1.13 (from ROC)	Admission, 24 h, 12 d	mRS ≥ 2 @ 3 months/mortality	3-mo poor: OR 1.18–1.22, AUC 0.82; Mortality: OR 1.17–1.25 (cNLR₁–₂/₁–₃ 1.21–1.23), AUC 0.86–0.90.	24 h and 12 d NLR and ΔNLR predicted poor outcome and mortality	High	*Brain & Behavior*, 2023
Li et al. (33)	China (Beijing)	102	Retrospective	IVT(rt-PA)	NLR, LMR	NEU/LYM; LYM/MON	NLR48h 5.69(AUC 0.79); LMR (48 h) = 2.48 (AUC 0.75)	Baseline (pre-IVT), 24 h, 48 h, discharge	ENI (24 h), mRS 0–1 at discharge	ENI – NLR₍24 h₎ 0.85 (*p* = 0.04, AUC 0.62); mRS 0–1 – NLR₍48 h₎ 0.64/LMR₍48 h₎ 1.50 (*p* = 0.01/0.02; AUC 0.79/0.75).	NLR24 h predicted ENI; NLR48 h and LMR48 h predicted favorable outcome	Moderate	*J Inflamm Res*, 2022
Xu et al. (61)	China (Shanghai)	286	Retrospective	IVT(rt-PA)	PLR	PLT/LYM	cut-off not stated	Baseline	mRS>2 @ 3 months	outcome OR2.220(1.245–3.957), death OR 2.825(1.050–7.601)	High PLR independently predicted poor functional outcome	Moderate	Front Neurology 2019
Ma et al. (45)	China (Nanjing)	190	Retrospective	IVT(rt-PA)	SII, SIRI, IPI	SII = PLT × NEU/LYM; SIRI = NEU × MON/LYM; IPI = CRP × SIRI	SII > 392.9 × 10^9^/L; SIRI > 1.298; IPI > 0.223	Admission	mRS ≥ 2 @ 90 days	AUC ≈ 0.70 (SIRI 0.720; SII 0.715; IPI 0.701); OR ≈ 1.09 (SIRI), 1.00 (SII), 7.11 (IPI)	High SIRI, IPI, and SII values are correlated with poor 90d outcomes in AIS patients undergoing intrave nous thrombolysis	High	Journal of Neuroinflammation 2023 20(1): 220
Wang et al. (47)	China (Suzhou)	717	Retrospective	IVT(rt-PA)	PIV	PLT × NEU × MON/LYM	283.84 (59% sensitivity and 62% specificity)	Baseline	mRS ≥ 2 @ 3 months	AUC 0.607 (0.560–0.654) OR ≈ 1.9–2.3(highest Q4 vs. Q1)	PIV independently predicted unfavorable 3-month mRS outcomes, showing a predictive performance comparable to SII, PLR, and NLR.	Moderate	Curr Neurovasc Res 2023 20(4): 464–471
Zhou et al. (62)	China (Chengdu)	278	Retrospective	IVT(rt-PA)	SII、NLR、PLR	SII = PLT × NEU/LYM; NLR = NEU/LYM; PLR = PLT/LYM	SII = 652.73; NLR = 3.57; PLR = 127.01	Baseline	mRS ≥ 3 @ 3 months	AUC 0.698 (SII); 0.694 (NLR);0.643 (PLR); Adjusted ORs: SII 1.001 (<0.001); NLR 1.268 (<0.001); PLR 1.009 (<0.001)	SII, NLR, and PLR independently predicted poor 90-day outcomes after IVT in AIS, with SII demonstrating the strongest predictive performance (AUC ≈ 0.70).	high	*PLOS ONE*, 2025
Zhao et al. (63)	China (Hebei)	281	Retrospective	IVT(rt-PA)	PT、SIRI、SII	SIRI = NEU × MON/LYM; SII = PLT × NEU/LYM	(ROC)PT: 10.85 s; SIRI: 0.926 × 10^9^/L; SII: 621.68 × 10^9^/L	Baseline	24 h END(NIHSS↑ ≥ 4);mRS ≥ 2 @ 3 months	OR: PT 1.833 (1.161–2.893); SIRI 2.166 (1.014–4.629); SII 1.002 (1.000–1.003); AUC: PT 0.669, SIRI 0.773, SII 0.787	PT, SIRI, SII independently predicted unfavorable 3-month outcomes; SIRI additionally predicted END.	High	*Clinical and Applied Thrombosis/Hemostasis*, 2023
Pektezel et al. (64)	Ankara Hacettepe University Hospitals	142	Retrospective	IVT(rt-PA)	NLR	NLR = NEU/LYM	Admission ≈ 4.1; 24 h ≈ 3.6 (ROC	Admission & 24 h post-tPA	NIHSS ≥ 4 or NIHSS ≤ 1;mRS ≤ 2 @ 3 months; Hemorrhagic complications.	Adm NLR AUC 0.576; 24 h NLR AUC 0.737(mRS ≤ 2); PH-2 AUC 0.931; *β* = −0.216 (*p* = 0.006).	Admission NLR: no predictive value; 24 h NLR↑ predicted poor 90-day outcome & sICH.	Moderate	*J Stroke Cerebrovasc Dis*, 2019
Cheng et al. (65)	China (Wenzhou)	381	Prospective	IVT(rt-PA)	NLR + blood glucose	NLR = NEU/LYM	High NLR ≥ 4.0 vs. Non-high NLR < 4.0 (based on median split)	Baseline (admission, within 24 h of onset)	(1) mRS ≥ 3 @ 3 months; (2) END (NIHSS increase ≥ 4 within 24 h); (3) 3-month mortality	Poor outcome OR 4.42 (2.13–9.16); END OR 4.81 (2.08–11.12); 3-month mortality OR 6.56 (1.92–22.40)	High NLR + hyperglycemia increased the risk of END, poor 3-month functional outcome and mortality;	Moderate	*Brain and Behavior*, 2020
Deng et al. (66)	China (Liaoning)	151	Retrospective	IVT(rt-PA)	NPAR, NLR, PLR, NPAR + NLR	NPAR = NEU%/ALB; NLR = NEU/LYM; PLR = PLT/LYM	ROC: NPAR 1.615; NLR 3.495; d NPAR + NLR sens 67.5%, spec 71.2%)	Baseline	mRS ≥ 2 @ 90 days	AUC: NPAR 0.72, NLR 0.71, combo 0.72 OR – NPAR 3.898 (1.079–14.087,); NLR 1.672 (1.056–2.647)	Baseline NPAR & NLR independently predict poor short-term outcome after IVT; NPAR highest AUC ≈ 0.72.	Moderate	*Frontiers in Neurology*, 2025
Li et al. (38)	China (Baoding)	762	Retrospective	IVT(rt-PA)	PLR; NLR; LMR; SII; SIRI; PIV	PLR = PLT/LYM; NLR = NEU/LYM; LMR = LYM/MON; SII = PLT × (NEU/LYM); SIRI = NEU × MON/LYM; PIV = NEU × PLT × MON/LYM	ROC cut-off not stated	Baseline	mRS>2 @ 3 months	AUC: PLR 0.61, NLR 0.71, LMR 0.61, SII 0.72, SIRI 0.63, PIV 0.57; OR = 1.00 (*p* = 0.013), 1.12 (0.029), 1.03 (<0.001), 1.33 (<0.001), 2.00 (0.038), 1.10 (0.081).	All six indices predicted poor 3-mo outcome; NLR & SII highest (AUC ≈ 0.71–0.72); PIV lowest (≈0.57).	Moderate	*International Journal of General Medicine* 2024
Weng et al. (41)	China (Wenzhou)	216	Retrospective	IVT(rt-PA)	SII	SII = (PLT × NEU/LYM)	545.14 × 10^9^/L (ROC)	Within 24 h after admission	mRS>2 @ 3 months	AUC 0.678 (0.001); multivariate OR = 3.953 (0.001)	SII was positively correlated with stroke severity& independently predicted poor 3-mo outcome; adding SII improved reclassification	high	*Clinical Interventions in Aging* 2021
Ma et al. (44)	China (Xinjiang)	63	Prospective	IVT(rt-PA)	SIRI; IPI	SIRI = NEU × MON/LYM; IPI = CRP × NEU/(LYM × ALB)	SIRI = 1.010 × 10^9^/L; IPI = 0.343	Baseline	mRS ≥ 3 @ 3 months	AUC: SIRI 0.685 (< 0.05); IPI 0.756 (0.05); Adjusted multivariate OR – SIRI 1.407 (0.044); IPI 1.306 (0.029)	SIRI & IPI↑ correlate with stroke severity; both independently predict poor 3-mo outcome; IPI higher AUC ≈ 0.76 vs. 0.69.	moderate	*International Journal of General Medicine* 2022
Chen et al. (18)	China (Hunan)	161	Retrospective	IVT(rt-PA)	SIRI	SIRI = NEU × MON/LYM	2.54 (ROC)	Admission/baseline within 24 h after stroke onset	mRS>2 @ 3 months	SIRI AUC = 0.7885; OR = 1.431 (0.028); NLR OR 1.331 (0.024)	SIRI >2.54 independently predicted poor 3-mo outcome; adding SIRI improved model AUC to 0.876 (ASPECTS+NIHSS+NLR + SIRI).	moderate	The Neurologist 2023

Comparative features of the included composite inflammatory indices are summarized in Table 3, detailing their constituent components, underlying biological mechanisms, and clinical applicability.

**Table 3:** Comparative summary of inflammation-based indices used in AIS (particularly IVT cohorts).

Index	Components/Formula	What it captures (biological meaning)	Typical prognostic targets in AIS/IVT	Representative evidence & key finding (concise)
NLR (Neutrophil-to-Lymphocyte Ratio)	NEU/LYM	Balance of innate neutrophil-driven inflammation vs. adaptive immunity; higher = pro-inflammatory tilt	90-day mRS poor outcome; early neuro-worsening; edema; mortality	High NLR consistently associated with worse 3-month function and higher mortality after stroke; also predicts edema progression.
SII (Systemic Immune-Inflammation Index)	(PLT × NEU)/LYM	Integrates platelets (thrombosis/activation) and neutrophil-dominant inflammation against lymphocyte count; more comprehensive than a single ratio	90-day (or longer) functional outcome (mRS), mortality; sometimes sICH	Widely used in stroke; SII independently predicts poor outcomes; formula widely standardized in neurology.
SIRI (Systemic Inflammation Response Index)	(NEU × MON)/LYM	Adds monocytes (myeloid activation/secondary inflammation) to neutrophil-lymphocyte balance	90-day poor outcome; mortality	Meta-analyses and clinical cohorts show SIRI is an independent predictor of 90-day poor prognosis in AIS, sometimes outperforming NLR-family ratios.
IPI (Inflammatory Prognostic Index)	(CRP × NLR)/Albumin (CRP/Alb ratio × NLR)	Couples acute-phase protein (CRP) and nutritional/anti-inflammatory reserve (albumin) with NLR; needs biochemistry + CBC	Functional outcome or mortality (evidence in stroke is emerging; widely used across other conditions)	Original definition: IPI = CRP × NLR/albumin; proposed as a stronger composite inflammatory score than single indices; being explored in vascular/neurologic cohorts.
PIV (Pan-Immune-Inflammation Value)	(NEU × MON × PLT)/LYM	A “pan-immune” composite from four CBC lines; emphasizes broad myeloid activation + thrombocytic component vs. lymphocytes	Mortality (short−/long-term), poor function; ICU/critical AIS risk stratification	In large retrospective datasets (e.g., MIMIC-IV AIS), higher PIV independently predicts short- and long-term mortality; formula standardized across reports.

## 4 Discussion

### 4.1 Clinical significance and principal findings

This systematic review demonstrates that inflammation-based hematological indices derived from routine complete blood counts—particularly NLR, SII, SIRI, and PIV—are consistently associated with 90-day functional outcomes following intravenous thrombolysis in patients with acute ischemic stroke (AIS). These indices capture the dynamic balance of systemic immune–inflammatory status, integrating the interactions among neutrophil-driven innate activation, monocyte-mediated secondary inflammation, and lymphocyte-regulated adaptive suppression. Their prognostic performance, typically reflected by AUC values of 0.70–0.80, indicates moderate-to-good discriminative capacity. Thus, these easily obtainable and cost-effective markers may complement existing clinical and neuroimaging predictors. Moreover, several studies reported that temporal changes in inflammatory indices within the first 24–48 h after thrombolysis were more informative than baseline values alone. This finding supports the emerging concept of continuous immune monitoring during the acute stage of AIS, in which dynamic immunologic trajectories may mirror early neurovascular injury and reperfusion-related stress. Although approximately 4,000 patients across 15 IVT-only studies were included, most available data originated from single-center retrospective Chinese cohorts. In contrast, international evidence remains scarce. Many non-Asian studies employed mixed IVT ± EVT designs or focused solely on conventional inflammatory markers (e.g., leukocyte count, C-reactive protein [CRP]) rather than composite indices such as NLR, SII, SIRI, and PIV. This geographical concentration restricts the generalizability of findings and contributes to heterogeneity in cutoff values and effect sizes across studies.

### 4.2 Mechanistic role of inflammation in acute ischemic stroke

**Figure 2:**
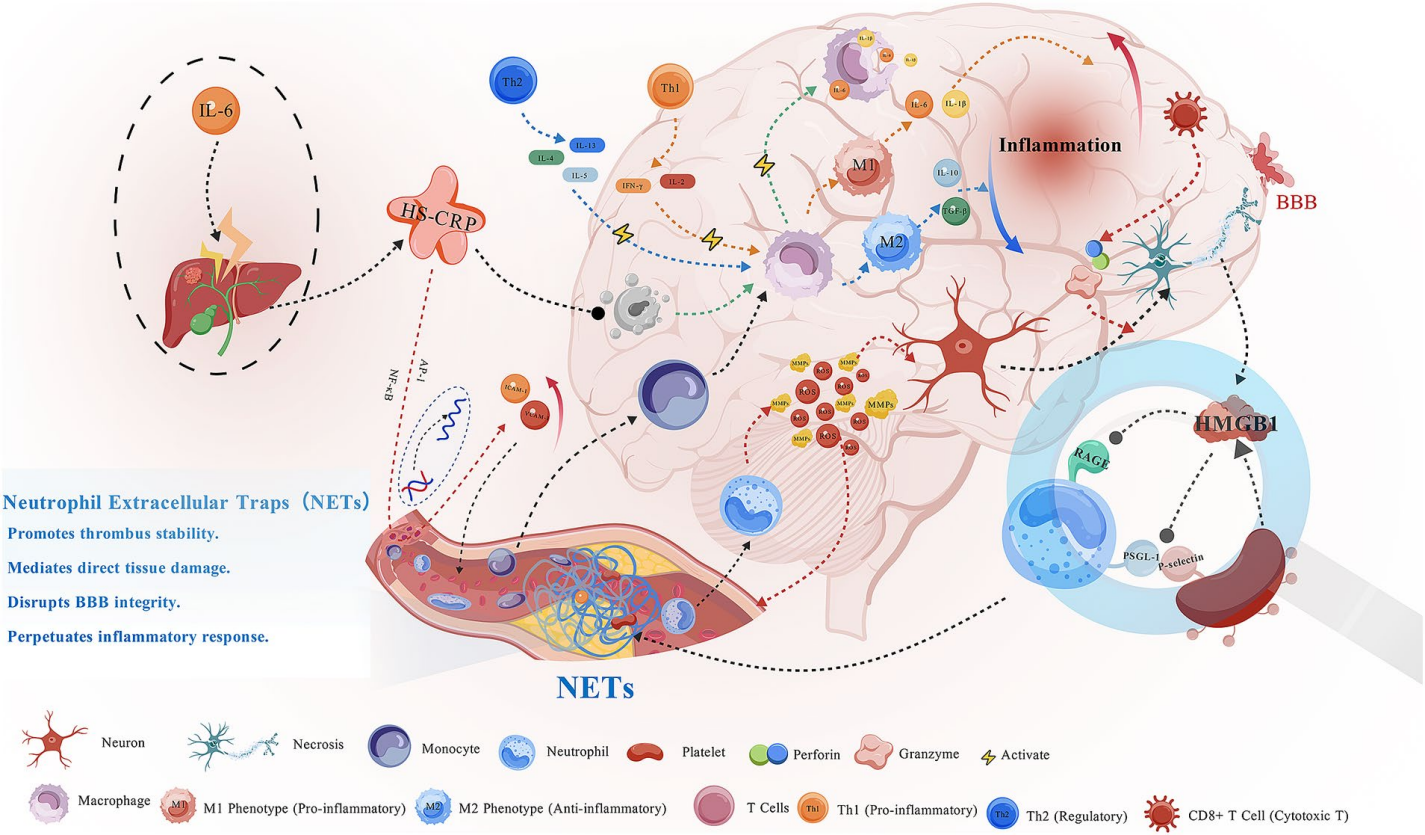
Systemic and neuroinflammatory responses after acute ischemic stroke and intravenous thrombolysis.

A growing body of evidence indicates that ischemic stroke is not merely a vascular occlusive event but rather a neuro-immune disorder involving complex interactions among glial cells, neurons, vascular endothelium, and extracellular matrix—collectively termed the neurovascular unit **(**17–19). Following ischemia, this intricate network becomes rapidly activated, triggering a cascade of inflammatory events (20). After cerebral ischemia, necrotic neurons release damage-associated molecular patterns (DAMPs) that engage Toll-like receptor (TLR) signaling on microglia and astrocytes, leading to their activation. Activated glial cells secrete large quantities of pro-inflammatory cytokines such as IL-1β, TNF-*α*, and IL-6, which not only exacerbate local neuronal injury but also stimulate hepatic synthesis of C-reactive protein (CRP) **(**21–23). High-sensitivity CRP (hs-CRP) serves as a marker of peripheral inflammation and further amplifies local injury via the complement cascade (24). The combined action of CRP and cytokines activates endothelial NF-κB/AP-1 signaling, up-regulating adhesion molecules (ICAM-1, VCAM-1) that facilitate leukocyte adhesion and transmigration across the vessel wall into ischemic tissue (22). Neutrophils then release neutrophil extracellular traps (NETs), reactive oxygen species (ROS), and proteases, which cause oxidative injury and tissue degradation (25, 26). Platelets, through P-selectin and high-mobility group box 1 (HMGB1), participate in inflammatory amplification and thrombosis, while monocytes differentiate into M1/M2 macrophages, orchestrating secondary immune regulation (27). T lymphocytes further modulate immune activity during the subacute phase, influencing both repair and reperfusion responses (22, 28). Ultimately, inflammatory mediators such as matrix metalloproteinase-9 (MMP-9) disrupt the blood–brain barrier (BBB), leading to edema, hemorrhagic transformation, and infarct expansion—thereby transforming focal vascular occlusion into widespread cerebral injury (29) (Figure 2). Collectively, these findings underscore that the inflammatory cascade in AIS represents a multicellular and multiphasic process, in which the interplay among immune cells, cytokines, and vascular elements not only aggravates ischemic damage but also provides a mechanistic foundation for prognostic biomarker development and targeted therapeutic intervention.

After acute ischemic stroke, necrotic neurons release damage-associated molecular patterns (DAMPs), which activate microglia and astrocytes via Toll-like receptor (TLR) signaling, inducing cytokines such as IL-1β, TNF-*α*, and IL-6. These mediators stimulate hepatic CRP production and endothelial activation, promoting leukocyte adhesion and blood–brain barrier (BBB) disruption. Neutrophils release extracellular traps (NETs), reactive oxygen species, and proteases, aggravating vascular injury. Activated platelets interact with neutrophils and monocytes through P-selectin and HMGB1, enhancing thrombosis and amplifying inflammatory signaling. Monocytes and macrophages (M1/M2) modulate inflammation and tissue repair, while T-cell subsets (Th1, Th2, CD8^+^) regulate adaptive immune responses. The interplay of these pathways perpetuates neuroinflammation and secondary ischemic injury. Composite hematological indices such as NLR, SII, SIRI, and PIV reflect this systemic–central inflammatory and thrombo-immune imbalance.

### 4.3 Neutrophil-to-lymphocyte ratio

#### 4.3.1 Formula


NLR=neutrophil count/lymphocyte count

#### 4.3.2 Biological rationale

NLR reflects the dynamic equilibrium between elevated neutrophils and reduced lymphocytes during systemic inflammation. In acute inflammatory states, neutrophils rise rapidly and release a variety of pro-inflammatory mediators that sustain tissue injury, whereas lymphocytes undergo apoptosis or redistribution due to stress responses, resulting in increased NLR (30, 31). This index thus integrates both enhanced innate activation and diminished adaptive immune regulation, characterizing the dual immunologic imbalance of stroke-related inflammation.

#### 4.3.3 Representative evidence and effect sizes

Wu et al. studied 259 AIS patients undergoing IVT and found that NLR at 24 h and 12 days were independent predictors of poor 90-day outcome (mRS ≥ 2) — adjusted OR 1.18 and 1.22, respectively — with AUC values of 0.815 and 0.820. NLR also predicted 90-day mortality (AUC = 0.86 and 0.90) (32). Similarly, Li et al. reported that 24 h NLR independently correlated with early neurological improvement (ENI) (OR 0.85, 95% CI 0.75–0.95, *p* = 0.04), and 48 h NLR (OR 0.64, 95% CI 0.49–0.83, *p* = 0.01) predicted favorable discharge outcome (mRS 0–1). ROC analysis showed 48 h NLR AUC = 0.79 (95% CI 0.70–0.88) (33).

#### 4.3.4 Limitations and clinical utility

NLR is nonspecific and easily confounded by infection, corticosteroid use, or stress hyperglycemia. Optimal cutoff values vary markedly across cohorts. Nevertheless, its simplicity, low cost, and immediate availability make NLR a valuable first-line inflammatory indicator for early risk stratification and therapeutic monitoring in AIS. Combining NLR with composite indices (e.g., SII, SIRI) may enhance predictive robustness.

### 4.4 Systemic inflammation response index

#### 4.4.1 Formula


SIRI=(neutrophil count×monocyte count)/lymphocyte count

#### 4.4.2 Biological rationale

SIRI integrates three principal immune-cell lineages—neutrophils, monocytes, and lymphocytes—thereby reflecting the interplay between acute inflammation intensity, chronic immune activation, and regulatory competence (34). Neutrophils represent early innate responses, monocytes mediate tissue repair and secondary inflammation, and lymphocytes denote adaptive immunosuppression. An elevated SIRI indicates disrupted equilibrium between pro- and anti-inflammatory pathways (35).

#### 4.4.3 Representative evidence and effect sizes

Chu et al. analyzed 240 patients with mild AIS receiving IVT and demonstrated that high SIRI was independently associated with poor 90-day outcome (mRS ≥ 2) (OR 2.94, 95% CI 1.81–4.78, *p* < 0.001). The AUC for SIRI alone was 0.714 (95% CI 0.65–0.77), rising to 0.773 when combined with clinical factors (36).

Li et al. (37) prospectively confirmed that elevated SIRI independently predicted adverse 90-day outcomes [OR 1.57, 95% CI 1.12–2.20, *p* = 0.010; (38)].

#### 4.4.4 Limitations and clinical utility

Most studies remain single-center and retrospective with modest sample sizes and limited temporal analyses. Future multicenter prospective studies are required to validate the dynamic prognostic utility of SIRI. Notably, SIRI shows minimal variation by sex or age and can be easily computed from blood counts, making it an ideal component for multivariable inflammation-based prediction models.

### 4.5 Systemic immune–inflammation index

#### 4.5.1 Formula


SII=(platelet count×neutrophil count)/lymphocyte count

#### 4.5.2 Biological rationale

SII combines platelet-mediated coagulation activity, neutrophil-driven inflammation, and lymphocyte-mediated immune modulation, thus providing a comprehensive representation of the inflammation–immunity–thrombosis triad (39). During the acute phase of AIS, platelet and neutrophil activation can precipitate microcirculatory obstruction, while reduced lymphocyte levels signify heightened stress-induced immunosuppression (40).

#### 4.5.3 Representative evidence and effect sizes

Multiple studies have confirmed that increased SII correlates with poorer functional recovery after AIS. Weng et al. reported SII AUC = 0.678 (95% CI 0.612–0.740); high SII (> 545.14 × 10^9^/L) was an independent risk factor (OR 3.95, 95% CI 1.70–9.18, *p* = 0.001) (41). Zhou et al. found SII AUC = 0.657 (95% CI 0.572–0.742); OR 2.92 (95% CI 1.42–5.99, *p* = 0.004) (42).

#### 4.5.4 Limitations and clinical utility

SII cutoffs vary widely—some studies use median splits, others ROC-based thresholds—hindering comparability. It is also susceptible to confounding by infection, diabetes, or stress responses. Nonetheless, SII requires only basic hematologic data, is reproducible, and remains one of the most promising prognostic indicators for post-thrombolysis outcomes in AIS.

### 4.6 Inflammation prognostic index

#### 4.6.1 Formula


IPI=C−reactive protein(CRP)×NLR/albumin(ALB)

#### 4.6.2 Biological rationale

The inflammation prognostic index integrates CRP, NLR, and albumin to simultaneously represent systemic inflammation, immune imbalance, and nutritional/anti-oxidative status, thereby reflecting inflammation-immunity-metabolism interactions (43). Elevated CRP signals heightened inflammatory activity, whereas decreased albumin indicates poor nutrition and reduced antioxidant capacity; their combination provides a global view of systemic inflammatory burden.

#### 4.6.3 Representative evidence and effect sizes

Ma et al. demonstrated that higher IPI was independently associated with poor 90-day outcome (mRS ≥ 3) (OR 1.31, 95% CI 0.89–1.95, *p* < 0.05) with AUC 0.756 (95% CI 0.604–0.826), outperforming SIRI (AUC 0.685) (44). Ma F et al. similarly showed IPI as an independent predictor (OR 7.11, 95% CI 1.66–30.46, *p* = 0.008; AUC 0.701, 95% CI 0.604–0.826), highlighting its moderate discriminatory value (45).

#### 4.6.4 Limitations and clinical utility

Calculation requires serum CRP and albumin assays, which may vary by methodology and metabolic status. Research in stroke populations remains limited. Nevertheless, IPI uniquely combines inflammatory and nutritional dimensions and may enhance prognostic models or serve in centers equipped for biochemical testing.

### 4.7 Pan-immune–inflammation value

#### 4.7.1 Formula


$$\begin{array}{l} \mathrm{PIV}=(\text{neutrophil count}\times \text{monocyte count}\times \text{platelet count})/\\ \text{lymphocyte count} \end{array}$$

#### 4.7.2 Biological rationale

PIV simultaneously captures the activity of four hematologic components, providing a global measure of immune-system activation. It reflects both myeloid-cell activation and platelet-mediated pro-inflammatory effects alongside lymphocyte-driven regulatory capacity (46).

#### 4.7.3 Representative evidence and effect sizes

Wang et al. examined 717 AIS patients treated with IVT and found that high PIV was significantly associated with poor 3-month outcome (Q4 vs. Q1 OR 2.23, 95% CI 1.23–4.04), AUC = 0.607 (95% CI 0.56–0.65), comparable to SII and SIRI (47).

Altuntaş et al. evaluated 1,039 severe AIS patients and reported PIV AUC = 0.921 (95% CI 0.90–0.94) for in-hospital mortality, superior to SII (AUC 0.887, *p* < 0.001) and comparable to the CALLY index (*p* = 0.385) (48).

#### 4.7.4 Limitations and clinical utility

Current PIV studies are few and heterogeneous; variations in sampling time, infection status, and population source affect reproducibility. Despite these limitations, its derivation solely from routine hematology and straightforward calculation make PIV a promising addition to mortality-risk and composite-inflammation models in severe AIS.

### 4.8 Composite inflammation index models and algorithmic optimization

The inflammatory response after stroke is a dynamic, multi-stage process, in which the predictive value of various indices may differ across time points. Integrating multiple composite inflammation markers into clinical prediction models can therefore enhance both accuracy and interpretability in individualized risk assessment.

#### 4.8.1 Model construction and predictive performance

Several recent studies have incorporated inflammation-based hematological indices into prognostic models for acute ischemic stroke (AIS).

For instance, Zhou et al. retrospectively analyzed 208 AIS patients and developed a multifactorial model combining SII with NIHSS, achieving an AUC of 0.826 (C-index 0.802), superior to any single variable. Elevated SII was identified as an independent predictor of poor 90-day outcome, and the joint inclusion of SII and clinical variables significantly improved predictive precision (42). Similarly, Chen et al. (49) *The Neurologist* evaluated 161 AIS patients receiving IVT and found that high SIRI (> 2.54) was an independent determinant of poor 3-month outcomes (AUC = 0.788). When SIRI, NLR, NIHSS, and ASPECTS were integrated into a nomogram, the AUC rose to 0.876, demonstrating strong discrimination and calibration (18).

Collectively, these studies underscore that composite inflammation indices provide incremental prognostic value beyond conventional variables and can substantially improve early identification of patients at risk for unfavorable functional recovery after thrombolysis.

#### 4.8.2 Emerging trends in model algorithms

Contemporary prognostic frameworks increasingly combine inflammation markers with clinical features (e.g., NIHSS, ASPECTS, and tPA dosage), imaging parameters (e.g., infarct volume, collateral circulation score), and laboratory indicators, yielding multidimensional predictive architectures.

While traditional multivariate logistic regression remains the mainstay, recent research has adopted machine-learning (ML) methods—including artificial neural networks (ANNs), random forests, support vector machines (SVMs), and deep-learning models—to enhance non-linear pattern detection (18, 50, 51).

Other studies have applied LASSO regression for feature selection in modeling early neurological deterioration (END) after thrombolysis (52), highlighting the potential of regularized and ML-based techniques when handling numerous correlated variables. Future clinical–biomarker hybrid models, integrating parameters such as NLR, SIRI, SII, and PIV with demographic, radiologic, and therapeutic factors, may yield superior predictive performance. Nevertheless, large-scale prospective multicenter studies with external validation remain essential to confirm generalizability and clinical robustness.

## 5 Current limitations and future perspectives

Although inflammation-related hematological models show promising potential for predicting functional outcomes after intravenous thrombolysis (IVT) in AIS, several limitations persist.

Most existing investigations are single-center retrospective studies lacking external validation and featuring limited sample sizes, thereby reducing model generalizability and statistical robustness. In addition, inconsistent sampling times (admission, 24 h, discharge) may introduce temporal bias, while the absence of multitime-point data constrains understanding of inflammatory evolution.

From a geographical perspective, nearly all included studies originate from Chinese single-center cohorts, whereas international IVT-only datasets remain scarce. Non-Chinese studies often employ mixed IVT ± EVT designs or focus solely on conventional inflammatory markers (e.g., leukocyte count, CRP), lacking systematic evaluation of composite indices such as NLR, SII, SIRI, and PIV. These disparities in region and design not only restrict external validity but also contribute to heterogeneity in cutoff definitions and effect magnitudes.

To advance this field, future investigations should emphasize:

Dynamic time-series modeling — constructing longitudinal prediction models based on sequential measurements of composite inflammation indices to capture temporal immune responses in the acute phase of AIS.
Multimodal integration — combining clinical, neuroimaging, and multi-omics (genomic, transcriptomic, proteomic) data to develop a comprehensive inflammatory prediction framework.
External and cross-ethnic validation — implementing large multicenter, multi-ethnic cohort studies to assess model transferability, improve interpretability, and enhance real-world clinical applicability.

## 6 Ethnic differences in inflammatory responses and future research outlook

Accumulating evidence indicates that the inflammatory response following acute ischemic stroke (AIS) is modulated by genetic background, environmental exposures, and lifestyle factors, leading to population-level variations in immune intensity, inflammatory cascades, and recovery trajectories (21, 53, 54). However, systematic investigations exploring ethnic or racial heterogeneity in these mechanisms remain extremely limited.

To date, most available studies have focused primarily on Han Chinese cohorts, whereas data on other ethnic groups—such as Yi, Zhuang, and Tibetan populations—are exceedingly scarce. This paucity of evidence restricts understanding of the universality versus specificity of inflammation-related prognostic markers across different genetic contexts. Epidemiological research has shown that ethnic disparities exist in baseline hematologic parameters, lipid profiles, and inflammatory mediator expression, for example, individuals of African descent typically exhibit lower neutrophil counts, while East Asian populations display greater systemic inflammatory sensitivity **(**55–57). These inherent differences may alter the baseline and interpretive thresholds of indices such as NLR, SII, and SIRI. Moreover, dietary patterns, obesity prevalence, infection burden, and chronic disease spectra vary considerably among populations and further shape systemic inflammatory status (58–60). Such heterogeneity underscores the necessity of incorporating ethnically diverse participants when developing and validating inflammation-based prognostic models.

### 6.1 Future research should prioritize

Inclusive multicenter studies that recruit patients from different ethnic and regional backgrounds to delineate the distribution patterns and prognostic implications of inflammation indices across populations.

Integration of genomic and transcriptomic technologies to identify race-associated immune pathway differences, including leukocyte subset ratios, cytokine expression profiles, and intracellular signaling cascades.

Large-scale prospective cohort studies implementing serial monitoring of inflammatory markers to elucidate gene–environment interactions governing post-stroke immune regulation.

Such efforts will not only clarify ethnic determinants of inflammatory dynamics but also support the creation of personalized, precision-based prognostic tools adaptable to diverse clinical settings.

## 7 Conclusion

Systemic inflammation plays a pivotal role in both the pathogenesis and recovery of acute ischemic stroke. Composite inflammation indices—such as SII, SIRI, IPI, and PIV—integrate multiple hematologic components, providing a comprehensive representation of immune–inflammatory balance and showing strong associations with post-thrombolysis functional outcomes. These indices offer clear advantages: they are economical, easily accessible, and highly reproducible, enabling practical application in early risk stratification and individualized management following intravenous thrombolysis. Future research should incorporate dynamic temporal analyses and multimodal predictive modeling to enhance prognostic precision and translational potential. Overall, the continued exploration of composite inflammation indices will help transition stroke management from empirical treatment toward precision prediction and personalized intervention, ultimately improving clinical outcomes for patients with acute ischemic stroke.

## Glossary

AUC: Area under the curve
AF: Atrial fibrillation
AIS: Acute ischemic stroke
ALB: Albumin
AP-1: Activator protein-1
ASPECTS: Alberta Stroke Program Early CT Score
BBB: Blood–brain barrier
BMI: Body mass index
BP: Blood pressure
CBC: Complete blood count
CI: Confidence interval
CRP: C-reactive protein
HS-CRP: High-sensitivity C-reactive protein
DAMPs: Damage-associated molecular patterns
END: Early neurological deterioration
ENI: Early neurological improvement
EVT: Endovascular thrombectomy
HMGB1: High mobility group box 1
HR: Hazard ratio
ICAM-1: Intercellular adhesion molecule-1
IL: Interleukin
IPI: Inflammation prognostic index
IVT: Intravenous thrombolysis
LMR: Lymphocyte-to-monocyte ratio
M1/M2: Macrophage phenotypes (pro-inflammatory/anti-inflammatory)
mRS: Modified Rankin Scale
MMP-9: Matrix metalloproteinase-9
MON: Monocyte count
NEU: Neutrophil count
NEU%: Neutrophil percentage
NETs: Neutrophil extracellular traps
NF-κB: Nuclear factor kappa-B
NIHSS: National Institutes of Health Stroke Scale
NLR: Neutrophil-to-lymphocyte ratio
NPAR: Neutrophil percentage-to-albumin ratio
NOS: Newcastle–Ottawa Scale
OR: odds ratio
PIV: pan-immune-inflammation value
PLR: platelet-to-lymphocyte ratio
PLT: platelet count
PRISMA: Preferred Reporting Items for Systematic Reviews and Meta-Analyses
PT: Prothrombin time
ROC: Receiver operating characteristic
ROS: Reactive oxygen species
rt-PA: Recombinant tissue plasminogen activator
SII: Systemic immune-inflammation index
SIRI: Systemic inflammation response index
cNLR: Change in neutrophil-to-lymphocyte ratio
sICH: Symptomatic intracranial hemorrhage
Th: T helper cell
TLR: Toll-like receptor
TNF-*α*: tumor necrosis factor-α
TOAST: Trial of Org 10,172 in Acute Stroke Treatment classification
VCAM-1: Vascular cell adhesion molecule-1
LYM: Lymphocyte count
